# Treatment of Tapeworm

**Published:** 1870-01

**Authors:** 


### Treatment of Tapeworm.

Dr. Johann Rulle, in an "Inaugural Dissertation," gives the following results, derived from the giving of certain components of the extracts of male-fern to twenty-nine patients affected with tænia: 1. Not only the filicic acid, but also its decomposition-products, which are soluble in alcohol, will destroy tapeworms. 2. One must be very cautious in concluding that tænia are destroyed while in the intestinal canal, as the absence of the ova of tapeworm does not always indicate the non-existence of the parisite. 3. The precipitate, thrown down on the addition of hydrochloric acid to extract of male-fern, previously treated with ammonia, is more active than filicic acid. Out of nine cases in which the acid alone was administered, there were two only in which the worm was completely expelled, while in two the agent was quite useless. With the precipitate, on the other hand, the worm was wholly discharged in four instances, and a completely negative result followed but once. 4. The pure filicic acid was administered in twenty-four instances without change of diet, and in fifteen where the diet was changed: in one of these cases the result was imperfect; and in a second, failed altogether. The hydrochloric acid precepitate failed in three instances in which the diet was unchanged; in nine other instances, in which the diet was restricted, there was not a single miscarriage. Hence the great importance of attending to the diet as a condition of success in the treatment of worms. 5. Filicic acid, given in the form of pill, removes tænia with the greatest certainty when it is combined with castor-oil. This increase in the action of the agent is to be ascribed, not to the solubility of the acid in the castor-oil, but rather to the drastic action of the latter remedy. 7. Drastics assist the cure of tapeworm, not only because they bring away the parasite, but also by their favoring the deeper penetration of the anthelmintic into the intestinal canal. 7. The acid is best administered in the impure form of pills, sixteen of which, containing 9.6 *grammes* of the remedy, should be made to serve for four doses. With two of these doses castor-oil is to be combined. This treatment should be preceded by a restriction in diet.—Schmidt's *Jahrbeucher*, No. 2, 1869, and *Druggist*, in *N. Y. Med. Journal.*

